# ENX-104: a selective and potent D_2/_D_3_ receptor antagonist enhances dopamine neurotransmission and reward responsiveness in translational rodent models

**DOI:** 10.1038/s41386-025-02287-w

**Published:** 2025-12-07

**Authors:** Krishna C. Vadodaria, Jordi Serrats, William Brubaker, Brian D. Kangas, Diego A. Pizzagalli, Dave S. Garvey, Vikram Sudarsan, Kimberly E. Vanover

**Affiliations:** 1 Engrail Therapeutics, San Diego, CA USA; 2 Harvard Medical School, McLean Hospital, Belmont, MA USA; 3 Noel Drury, M.D. Institute for Translational Depression Discoveries, University of California, Irvine, CA USA; 4 DSG Pharma Consulting LLC, Little Rock, AR USA

**Keywords:** Target validation, Pharmacology

## Abstract

Anhedonia, characterized by a diminished reactivity to pleasurable stimuli, is a core symptom across multiple neuropsychiatric disorders, including major depressive disorder. Disruptions in dopaminergic neurotransmission and dysfunction within the mesolimbic dopaminergic circuitry are key contributors to reward processing deficits. We hypothesized that low receptor occupancy–mediated antagonism of presynaptic inhibitory D_2_/D_3_ receptors could enhance dopaminergic neurotransmission and, in turn, improve reward responsiveness, a behavioral phenotype implicated in anhedonia. For this purpose, we developed ENX-104, a highly selective and potent D_2_/D_3_ receptor antagonist with favorable CNS pharmacokinetics, characterized by high brain penetrance and rapid plasma clearance. In preclinical studies, ENX-104 produced sustained increases in dopamine levels in the nucleus accumbens in rats. In the Probabilistic Reward Task (PRT), a preclinical model of reward responsiveness reverse-translated for rats from human studies designed to objectively quantify subdomains of anhedonia, ENX-104 enhanced reward responsiveness at low doses corresponding to approximately 10–50% D_2_/D_3_ receptor occupancy. Predictably, higher doses (65–80% receptor occupancy) were associated with antipsychotic-like effects in the rat conditioned avoidance response assay, while extrapyramidal side effects, such as catalepsy, emerged only at much higher occupancies ( > 80% receptor occupancy). Our integrated pharmacokinetic-pharmacodynamic modeling suggests that a once-daily oral dosing regimen of ENX-104 could enable low D_2_/D_3_ receptor occupancies, potentially offering a novel therapeutic approach for the treatment of psychiatric conditions characterized by deficits in reward responsiveness.

## Introduction

Anhedonia, the lack of reactivity to previously rewarding stimuli, is a core symptom that is experienced by 60-70% of major depressive disorder (MDD) patients. It predicts poor disease course yet remains largely unresponsive to currently approved antidepressants [1, 2]. Anhedonia in MDD is, in part, characterized by reduced reward-related striatal activation and dysfunctional reward learning, reflecting deficient dopaminergic signaling [3, 4]. Indeed, depletion of dopamine can induce symptoms of depression and anhedonia [5], whereas increasing dopaminergic neurotransmission has been shown to alleviate symptoms of depression and anhedonia [6]. However, the construct of anhedonia, like depression, is heterogeneous and multifaceted in humans and encompasses both impaired hedonic capacity and motivational deficits as well as reduced reward learning [7, 8]. While dopamine may not directly mediate the experience of pleasure, per se, dopaminergic neurotransmission has been linked to reward-driven motivational processes and reward learning [9]. Dopamine plays a role in reward-related behavior and motor function, exerting its effects via distinct neural circuits [9]. The mesolimbic pathway includes projections from the ventral tegmental area (VTA) in the midbrain to the ventral striatum, including the nucleus accumbens. This pathway is also referred to as the reward pathway and is central to motivation and reward-related behavior and dopaminergic activation in it is associated with reward encoding and incentive motivation [10–12].

Dopamine exerts its effects by binding to G-protein coupled dopamine receptors, categorized into D_1_-like (D_1_, D_5_) and D_2_-like (D_2_, D_3_, D_4_) families. D_1_-like receptors are primarily Gs-coupled and excitatory, while D_2_-like receptors are Gi-coupled and inhibitory. Both D_2_ and D_3_ receptors are expressed pre- and postsynaptically. At presynaptic sites, they function as autoreceptors regulating dopamine release through feedback inhibition. D_2_ receptors exist in two isoforms, the long form (D_2L_) and short form (D_2S_) and of the two, D_2L_ is the most abundantly expressed in the brain [13]. D_2L_ receptors are found at both pre- and postsynaptic sites, whereas D_2S_ receptors show presynaptic enrichment with substantially lower overall expression [13]. Both D_2_ and D_3_ receptors are expressed at postsynaptic sites and at presynaptic terminals. D_2_/D_3_ receptors are particularly abundant at presynaptic sites, where they act as autoreceptors to mediate feedback inhibition of dopamine neuron activity, thereby modulating extracellular dopamine levels. Notably, D_3_ receptors are enriched in the limbic system and striatal regions, areas central to reward processing and motivation, making them a target of growing interest in neuropsychiatric research.

D_2_ receptor antagonists have long been used as antipsychotic agents, as they dopaminergic neurotransmission. Yet, studies indicate the potential of low doses of a prototypical D_2_/D_3_ receptor antagonist amisulpride, in the treatment of depression and anhedonia [14–16], and clinical proof-of-mechanism imaging studies show that a low dose of amisulpride rescued the blunted reward-related striatal activation in unmedicated depressed individuals [17–19]. Thus, we hypothesized that preferential antagonism of presynaptic inhibitory D_2_/D_3_ receptors, achieved through low receptor occupancy, could enhance dopaminergic neurotransmission and, in turn, improve reward responsiveness.

We developed ENX-104, for low-dose administration to preferentially block presynaptic D_2_/D_3_ autoreceptors and enhance dopaminergic neurotransmission in the reward circuitry. ENX-104 is structurally related to nemonapride, a substituted benzamide historically used as an antipsychotic in Japan and South Korea at relatively high doses and with a thrice-daily oral regimen [20, 21]. ENX-104 is a deuterated derivative of the (cis-S,S) enantiomer of nemonapride, designed for a more favorable pharmacokinetic profile. In this study, using preclinical in vitro and in vivo assays, we characterized the pharmacology, pharmacokinetic and pharmacodynamic profile of ENX-104. To assess its therapeutic potential as an anti-anhedonic agent, we utilized the probabilistic reward task (PRT). The PRT was originally developed to objectively quantify reward responsiveness in clinical populations with anhedonia [22]. In this computerized task, humans make rapid visual discriminations in which correct responses in the presence of one stimulus are three times more likely to result in reward (rich stimulus) than correct responses in the presence of the other (lean stimulus). As shown across numerous studies [23–27], heathy control participants readily develop the adaptive response bias for the more richly rewarded stimulus, whereas participants with anhedonia are often observed to have a blunted response bias which correlates with current and predicts future anhedonia [23, 25]. Given its utility, the PRT has been chosen as recommended task to probe the positive valence systems by the Research Domain Criteria (RDoC) in its most recent revision (NIMH, 2016). More recently, the PRT has been reverse-translated using touchscreen technology to study reward responsiveness in rodents and nonhuman primates [26]. In the present stud, we used the rat PRT [28] to determine the ability of ENX-104 to enhance reward responsiveness, which would be evident in drug-induced increases in response bias for the more richly rewarded stimulus [29, 30]. Additionally, we evaluated antipsychotic-like effects and potential motor side effects at higher doses, using the conditioned avoidance response and catalepsy assays in rodents, respectively. To contextualize the dose-dependent effects of ENX-104, we integrated pharmacokinetic-pharmacodynamic (PK-PD) data to identify a target D_2_/D_3_ receptor occupancy range associated with antianhedonic efficacy. Together, our data support a once-daily dosing regimen of ENX-104 at low receptor occupancies, offering a novel therapeutic approach for potentially treating psychiatric conditions such as major depressive disorder (MDD), in which deficits in reward responsiveness are prominent.

## Materials and methods

### In vitro pharmacology

Binding and functional activity of ENX-104 at human dopaminergic receptors were tested using recombinant mammalian cell lines expressing respective receptors. Binding assays were conducted using radiolabeled reference tracers and compared against reference comparators. ENX-104 was tested at eight concentrations in duplicate to test for radioligand binding, agonist, or antagonist functional activity. Agonist activity was expressed as a percent of the activity of the reference agonist at its EC_100_ concentration or percentage of the inhibition of the reference agonist at its EC_80_ concentration for antagonist activity. Data were averaged values derived from multiple experiments. Experimental details are listed in Supplemental Methods.

### Pharmacokinetic studies

The pharmacokinetic (PK) distribution and brain penetration of ENX-104 were determined following oral administration to male Sprague Dawley rats (from Charles River) at Frontage laboratories. Rats (n = 3 per group) received a single oral (per os, po) dose of 2.5 mg/kg of ENX-104 and blood and brain samples were collected at 1, 2, 4, 8 and 24 h post-dose. ENX-104 plasma and brain concentrations were determined by liquid chromatography-tandem mass spectrometry. Experimental details on plasma/brain collection, tissue processing, and analyses are in Supplemental methods.

### D_2_/D_3_ receptor occupancy studies

Sprague Dawley rats from Charles River (Margate) were obtained and on the day of testing, animals were dosed po with either vehicle (0.5% methylcellulose) or ENX-104 at a single dose (2.5 mg/kg) or the positive control olanzapine (10 mg/kg) (n = 5/group). Rats were euthanized 1, 2, 4, 8 or 24 h after ENX-104 administration or 1 h after vehicle or olanzapine administration.

A post-mortem blood sample (~5 ml) was taken by cardiac puncture and placed into K/EDTA tubes (32.332, Sarstedt) and processed and stored at −80 °C for analysis. Whole brains were removed, rinsed with saline and blot dried. Striata were dissected out and weighed before being frozen on dry ice.

The value for specific binding in disintegrations per minute (DPM) was generated by subtraction of mean non-specific binding from mean total binding for each animal. Data were calculated as mean specific binding, as a percentage of the vehicle-treated control taken as 100% corresponding to a mean receptor occupancy as 0%. All data were square root transformed and analyzed by one-way analysis of variance (ANOVA), and the sample size was determined to appropriately power the study. ENX-104 was compared to vehicle by Dunnett’s test. Olanzapine was compared to vehicle by multiple t-test. Means were back-transformed to original units and adjusted for difference between vehicle and olanzapine groups. Experimental details can be found in Supplemental Methods.

### In vivo microdialysis

Experiments were carried out in male Sprague Dawley rats (Charles River, UK) (n = 7–8/group). For the surgery, rats were anaesthetized with isoflurane in O_2_ delivered via an anesthetic unit (Burtons Medical Equipment Ltd, UK). A dual-probe study was conducted whereby each rat had two concentric microdialysis probes (CMA 12 Elite probes, CMA Sweden) stereotaxically implanted into the prefrontal cortex (co-ordinates: AP + 3.2 mm; ML + /−2.5 mm relative to bregma; DV-4.0 mm relative to the skull surface, 2 mm tip) and nucleus accumbens (co-ordinates: AP + 2.2 mm; ML + /-1.5 mm relative to bregma; DV-8.0 mm relative to the skull surface, 2 mm tip). Coordinates were taken from [22]. Microdialysis experiments were performed the day after surgery. Microdialysate samples were collected from freely-moving rats at 30 min intervals for a baseline period of 120 min before administration of vehicle or drug. Samples were then collected for 8 h after drug treatment at intervals of 30 min.

The povehicle was 0.5% methylcellulose (400 cP, Sigma-Aldrich, Lot SLCB9094, pH 7.1) and a dose volume of 5 ml/kg po was used. The intraperitoneal (IP) vehicle was 0.9% saline (pH 5.6) and a dose volume of 2 ml/kg ip was used. All drug solutions were prepared on the day of use. ENX-104 (1 mg/kg and 2.5 mg/kg) was formulated in the po vehicle. D-Amphetamine sulfate (0.3 mg/kg) (Tocris, Batch No. 7 A/222903) formulation was prepared as a solution suitable for ip dosing.

Microdialysis data were log transformed. Baseline was defined as the geometric mean of the four pre-treatment samples (i.e., at −90, −60, −30 and 0 min). Data were log transformed and analysis was by robust regression using M estimation, Huber weighting, using the default parameter c = 1.345 with treatment as a factor and log(baseline) as a covariate. Each time was analyzed separately, together with means during each of the eight h after dosing and the overall 0-8 h after dosing. Comparisons to vehicle were by Williams’ test for ENX-104 and by the multiple t-test for d-amphetamine, and ENX-104 + d-amphetamine. Comparisons to d-amphetamine alone and ENX-104 alone for ENX-104 + d-amphetamine were by the multiple t-test and sample size was determined to appropriately power the study. One animal was excluded from the analysis for the nucleus accumbens due to poor chromatography. Experimental details are in Supplemental Methods.

### Probabilistic reward task

Adult male Sprague Dawley rats (n = 8/group) obtained from Charles River Laboratories (Wilmington, MA) weighing between 250 and 300 g were used. Rats in the PRT study were group housed as a form of environmental enrichment; however, to match previous studies using this protocol, nothing else was provided. Utilizing touchscreen apparatus and previously published protocols rats were trained to discriminate a long versus short time via differential responding on one of two virtual levers (see Fig. 4A) [28, 31, 32]. Following line-length discrimination training, 3:1 rich/lean probabilistic reinforcement schedules were introduced such that 60% of correct responses to one of the line lengths (e.g., long line = rich stimulus) and 20% of correct responses to the other line length (e.g., short line = lean stimulus) were rewarded. Rich/lean line assignment was counterbalanced across subjects and 50 trials of each trial type were presented in a quasi-random sequence.

Following the establishment of response biases under probabilistic contingencies, an acute drug testing protocol was arranged that included intermittent maintenance sessions in which correct responses on all trials were reinforced, control sessions in which 3:1 (60%:20%) rich/lean probabilistic contingencies were arranged and, no more than once per week, a drug testing session in which vehicle or a dose of ENX-104 (0.5, 1, or 2.5 mg/kg) was tested by administering po, 4 h prior to a 3:1 (60%:20%) probabilistic session. Doses of ENX-104 were tested in a mixed order across subjects using a Latin Square design. Vehicle (0.5% methylcellulose) and all doses of ENX-104 were tested in all 8 rats. ENX-104 doses and pretreatment interval were chosen based on pharmacokinetic data. Prior to ENX-104, amisulpride (1, 5, 50 mg/kg sc) was tested in an independent sample under otherwise identical conditions in the PRT.

### Data analysis

The PRT yields two primary dependent measures: response bias and discriminability. These can be quantified by examining the number of correct and incorrect responses in rich and lean trial types using, respectively, log *b* and log *d* equations derived from signal detection theory [26]:

$$\log b=0.5* \log \left(\frac{\left({{Rich}}_{{Correct}}+0.5\right)* \left({{Lean}}_{{Incorrect}}+0.5\right)}{\left({{Rich}}_{{Incorrect}}+0.5\right)* \left({{Lean}}_{{Correct}}+0.5\right)}\right)$$

$$\log d=0.5* \log \left(\frac{\left({{Rich}}_{{Correct}}+0.5\right)* \left({{Lean}}_{{Correct}}+0.5\right)}{\left({{Rich}}_{{Incorrect}}+0.5\right)* \left({{Lean}}_{{Incorrect}}+0.5\right)}\right)$$

High bias values are produced by high numbers of correct responses during rich trials and incorrect responses during lean trials, which increase the log *b* numerator. High discriminability values are produced by high numbers of correct responses during both rich and lean trials, which increase the log *d* numerator. (0.5 is added to all parameters to allow log transformation in cases of no errors). Given the a priori hypotheses discussed above of low doses of ENX-104 increasing reward responsiveness (putatively due to presynaptic autoreceptor blockade) but high doses decreasing reward responsiveness (putatively due to postsynaptic blockade), log *b* data were subjected to general linear model tests of within-subjects contrast in which the quadratic term was specifically evaluated (an inverted U-function was expected). All other data (log *d*) were subject to repeated measures ANOVA. For accuracy and reaction time, the repeated measures factor trial type (rich vs. lean) was added to the model. When appropriate, ANOVAs were followed by post-hoc tests (one-way: Dunnett’s multiple comparisons test; two-way: Bonferroni multiple comparisons test) to evaluate the statistical significance of stimulus type and drug treatment. Given a priori hypotheses (see below), paired t-tests and Cohen’s *d* were also used to evaluate, respectively, the statistical significance of differences in log *b* values and effect sizes following vehicle treatment and each individual dose of ENX-104. Effect sizes (Cohen’s *d* values) were interpreted using established conventions: small (*d* = 0.20), medium (*d* = 0.50), and large effect (*d* > 0.80). Sample size was determined to appropriately power the study and all statistical analyses were conducted using GraphPad Prism 10 Software (San Diego, CA, USA). Further details on testing apparatus, experimental procedure, and data analyses are in Supplemental Methods.

### Conditioned avoidance response

The Conditioned Avoidance Response (CAR) Test has been shown to be a very reliable animal model for screening antipsychotic drugs. In the CAR paradigm, an animal is trained to respond to a conditioned stimulus (auditory and visual) by negative reinforcement (foot shock). Numerous studies have shown that typical and atypical antipsychotic drugs selectively suppress avoidance response in CAR, thus making it one the preferred assay to screen potential antipsychotic compounds. Adult, male Wistar rats from Envigo (Indianapolis, IN) were used in this study (n = 10/group) and randomly assigned across the treatment groups. The experiments were conducted during the animal’s light cycle phase. ENX-104 (0.5, 2.5 and 5 mg/kg) was formulated in 0.5% Methyl Cellulose in water and administered orally at a dose volume of 1 ml/kg 4 h prior to test.

The CAR apparatus consists of a two-way shuttle box with infra-red (I/R) detection housed in sound-attenuating chambers (Med Associates). The two-way shuttle boxes have stainless steel grid floors and are partitioned by a guillotine door. Programs are run through Med-PC version IV software. Rats were trained to avoid a foot shock following presentation of a light or tone. Rats were placed in the CAR two-compartment shuttle box and presented with a conditioned stimulus (CS; light and tone), followed by an aversive unconditioned stimulus (US; foot-shock of 0.65 mA). Each rat goes through 20 trials with a variable ITI (20 – 60 sec). After several weeks of training in the CAR chambers, rats that passed the testing criterion of performing 16 -20 avoidance responses for three days in a row are included in the study and testing commenced. CAR testing consists of the same procedure as CAR training. Baseline was measured over three days prior to drug testing. Following the test, the rats were given one week washout between tests. The measures obtained from this test are:

#### Avoidance response

If the rat moved from one compartment to the other during the cued stimulus (CS) presentation and prior to foot-shock delivery. Decreased avoidance responding is the typical signature of an efficacious dose of an antipsychotic.

#### Escape failure

If the rat failed to move into the other compartment during the 20 s footshock.

Avoidance response data are presented as percent of avoidance responses based on the three baseline responses prior to drug test. Escape failures were expressed as the total number of failures during the test session. Sample size was determined to appropriately power the study and Dunnett’s post-hoc comparisons when appropriate. Escape failures were analyzed by Kruskal-Wallis nonparametric analysis followed by Dunnett’s post-hoc comparisons when appropriate. Results are reported as mean ± SEM. An effect was considered significant if p < 0.05.

### Catalepsy

Adult Sprague-Dawley male rats were obtained from Charles River (Margate) and assigned randomly across treatment groups (n = 8/group): Vehicle, haloperidol (positive control; Tocris, Product Code 0931; Lot: 4B/263582), and ENX-104 at 1, 2.5, or 10 mg/kg. ENX-104 was prepared in 0.5% methylcellulose and dosed orally. All compounds were formulated on the day of dosing and administered using a dose volume of 5 ml/kg.

On the day of the test, animals were dosed with vehicle (po), ENX-104 (1, 2.5 and 10 mg/kg, po) or haloperidol (0.82 mg/kg, ip; ED_75_ dose). Animals were tested individually for catalepsy at 90, 180 (data not shown) and 240 min post-dose by gently placing each paw in turn on a large rubber bung (42 mm high, 45 mm wide at upper surface). A score of 1 was given for each paw which remained in position for 15 s, giving each rat a maximum score of 4. The duration the paw remained in position was recorded (maximum latency score 15 s on each trial) and total latency for paw withdrawal was determined giving each rat a maximum total latency of 60 s. Sample size was determined to appropriately power the study and for statistical analyses, the total catalepsy score and total latency were compared to vehicle and haloperidol by exact Wilcoxon rank sum tests. Raw means and standard errors were calculated. p < 0.05 was considered statistically significant.

#### Statement of ethics for animal experimentation

All animal experimentation was performed in strict accordance with ethical standards of the respective institutions as well as guidelines provided for humane and ethical treatment of animals. In vivo microdialysis experiments were performed in accordance with Home Office Guidelines and licensed under the Animals (Scientific Procedures) Act 1986 (Project License P58B4DA70). The PRT protocol was approved by the Institutional Animal Care and Use Committee at McLean Hospital and in accordance with guidelines provided by the Committee on Care and Use of Laboratory Animals of the Institute of Laboratory Animals Resources, Commission on Life Sciences (National Research Council, 2011).

## Results

### ENX-104 is a selective and potent D2/D3 receptor antagonist

**Fig. 1:**
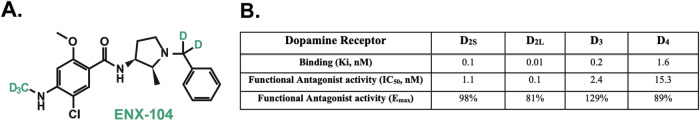
ENX-104 in vitro pharmacology at human receptors. **A** Structure of ENX-104 with deuterium (D) substitutions shown in green. **B** ENX-104 in vitro pharmacology at human dopamine D_2S_ (short form), D_2L_ (long form), D_3_ and D_4_ receptors showing binding affinity (Ki), potency as a functional antagonist (IC_50_) and efficacy as a functional antagonist (E_max_).

ENX-104 (C_21_H_21_D_5_ClN_3_O_2_) is a deuterated molecule with the molecular weight of 392.94 g/mol (Fig. 1A). ENX-104 displayed potent binding at all the D_2_-like inhibitory dopamine receptors tested (D_2S_, D_2L_, D_3_, and D_4_). The most potent binding and full antagonist activity was observed for D_2L_, affinity of 0.01 nM (Ki), and 0.1 nM (half-maximal inhibitory concentration [IC_50_]). ENX-104 also bound strongly to and displayed potent antagonist activity at D_2S_ (Ki = 0.1 nM, IC_50_ = 1.1 nM) and D_3_ (Ki = 0.2 nM, IC_50_ = 2.4 nM) receptors. Compared to D_2L_, ENX-104 showed weaker antagonist activity at the D_4_ receptor (Ki = 1.6 nM, IC_50_ = 15.3 nM) (Fig. 1B). ENX-104 exhibited over a hundred-fold weaker affinity for other receptors evaluated in a broad in vitro screening assay (data not shown). Thus, ENX-104 displayed selective and potent antagonism at D_2_/D_3_ receptors with limited off-target activity.

### ENX-104 displays brain enrichment, retention, and target engagement

**Fig. 2:**
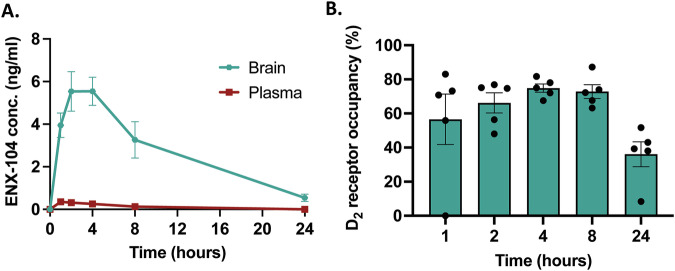
Pharmacokinetic profile and receptor occupancy of ENX-104. **A** Pharmacokinetic profile of ENX-104 in the rat after a single 2.5 mg/kg oral dose. Shown are ENX-104 levels (ng/ml) in the brain (green) and plasma (red) at five time points tested, over 24 h (n = 3/group). **B** D_2_/D_3_ receptor occupancy time course data at five time points over 24 h (n = 5/group) after a single oral dose of ENX-104 (2.5 mg/kg); dots representing data from individual animals. Data are presented as means ± SEM (error bars).

Following administration of an oral dose of 2.5 mg/kg, ENX-104 exhibited rapid peripheral metabolism, with a short plasma half-life and significant brain enrichment and retention over the course of 24 h. For rat PK studies, we focused on 2.5 mg/kg dose levels to gain accurate insights especially at 24 h post dose. Given the rapid metabolization of ENX-104, at lower dose levels (0.5 mg/kg) plasma drug levels drop below the detection limit at the 24 h time point (data not shown), even though brain levels are detectable. To more accurately calculate drug and ratios of brain:plasma levels over 24 h, we chose the 2.5 mg/kg dose for pharmacokinetic and receptor occupancy studies. Although plasma concentrations started decreasing by 2 h, brain concentrations were at maximum approximately 2-4 h post-dose. ENX-104 demonstrated excellent penetration into the central nervous system. Brain ENX-104 levels were higher than plasma levels at 1, 4, 8, and 24 h after dosing, with an approximately 10-fold enrichment at the 4 h timepoint (Fig. 2A).

At this dose, ENX-104 exhibited significant striatal D_2_/D_3_ receptor occupancy at 1, 2, 4, and 8 h (averages at 1 h, ~59%; 2 h, ~67%; 4 h, ~75%; and 8 h, ~73%) (Fig. 2B). Maximum receptor occupancy was observed 4 h (~ 75%) post dosing. Notably, while D_2_/D_3_ receptor occupancy was also observed 24 h post-dosing but lower relative to peak levels, indicating the reversibility of ENX-104 binding to the D_2_/D_3_ receptor within 24 hours. Thus, striatal D_2_/D_3_ receptor occupancy for ENX-104 mirrored the brain concentrations observed across the study. In contrast, plasma concentrations of ENX-104 reached a plateau in advance of peak of D_2_/D_3_ receptor occupancy. ENX-104 showed 4 to 40-fold brain:plasma ratios over the course of 24 h. Thus, ENX-104, with rapid plasma clearance and excellent blood-brain-barrier penetration after oral dosing, exhibits a favorable PK profile for a CNS drug.

### ENX-104 increased dopamine levels in the reward circuitry

**Fig. 3:**
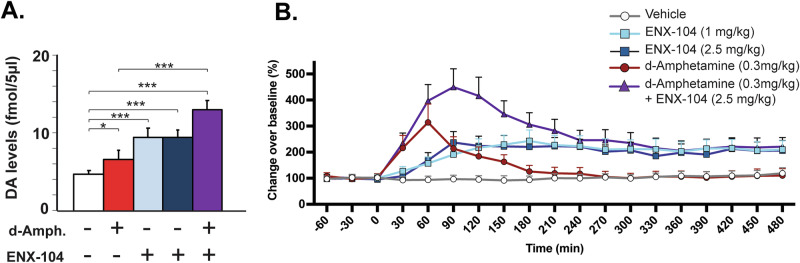
Dopamine levels in the nucleus accumbens. Animals were administered vehicle (white, ip and po), d-amphetamine (positive control, 0.3 mg/kg ip; red), ENX-104 (1 mg/kg po, light blue), ENX-104 (2.5 mg/kg po, dark blue), d-amphetamine+ENX-104 (2.5 mg/kg po) (purple) and dialysate collected at 30 min intervals, up to 8 h post dose (n = 7-8/group). **A** Shown are average dopamine levels over an 8-hour period post dosing ± SEM, *p < 0.05, ***p < 0.001. **B** Time course of dopamine reveals significant and sustained effects of ENX-104 (1 and 2.5 mg/kg dose levels) up to 8 h post dose. Data are presented as adjusted means (from log-transformed data) *±* SEM. Statistically significant differences between groups per time point in are shown in Supplementary Fig. S1.

To test whether ENX-104 treatment enhanced dopamine in the reward circuit (presumably, via blockade of D_2_/D_3_ autoreceptors), dopamine release was directly measured using in vivo microdialysis in freely moving rats following oral administration. As hypothesized, ENX‑104 significantly increased extracellular dopamine in both the nucleus accumbens and the prefrontal cortex (Fig. 3, Supplemental Fig. S1). In the nucleus accumbens, both doses (1 and 2.5 mg/kg) resulted in a significant and sustained increase averaged over the entire 8-hour sampling period (Fig. 3A, B). ENX-104 (1 mg/kg) produced a maximal increase (159% compared to controls; p < 0.001). ENX-104 at 2.5 mg/kg produced a similar pattern with a maximal increase (146% compared to controls; p < 0.001) observed at 90 min post-dose. In the prefrontal cortex, only the 2.5 mg/kg dose level resulted in a transient but significant change in dopamine levels (Supplemental Fig. S1). d-Amphetamine (0.3 mg/kg ip), used as a positive control, significantly increased dopamine in the nucleus accumbens as expected (Fig. 3A, B) and transiently in the prefrontal cortex (Supplemental Fig. S1). When ENX‑104 (2.5 mg/kg, po) was administered together with d-amphetamine (0.3 mg/kg, ip), the increases in dopamine in the nucleus accumbens were significantly greater as compared to controls (366%, p < 0.001). At the time of peak effect (1-2 h), average dopamine levels in the combination group were significantly greater than ENX‑104 alone (126%, p < 0.001) or d‑amphetamine alone (118%, p < 0.001) (Fig. 3B). This provides mechanistic proof that blockade of autoreceptors together with a dopamine releasing agent like d-amphetamine results in further increase in extracellular dopamine levels. Further, in comparison to d-amphetamine, which only transiently increased dopamine (~2.5 h post dose), ENX-104 increased dopamine to physiologically relevant levels and over a period of ~8 h within the reward circuit.

### ENX-104 enhanced reward responsiveness in the PRT

**Fig. 4:**
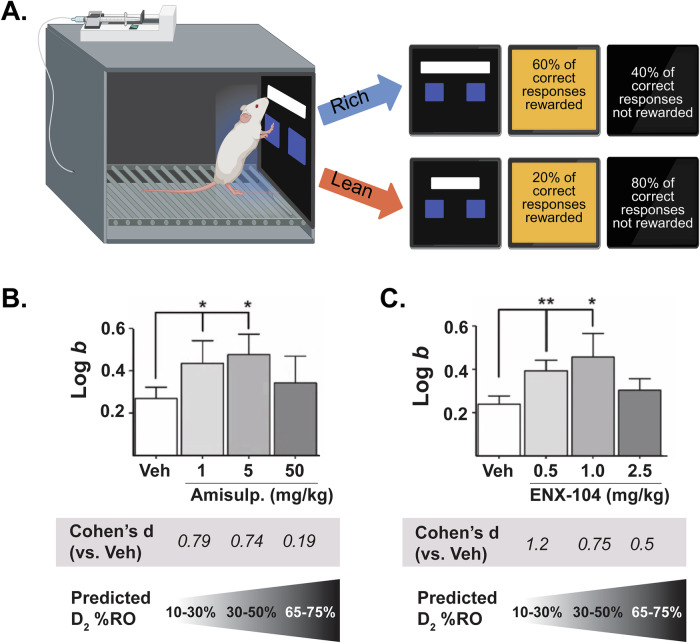
Anti-anhedonic effects in the rodent Probabilistic Reward Task (PRT). **A** Schematic of the touchscreen PRT testing conditions. In the PRT, doses of amisulpride and ENX-104 targeting low but not high D_2/3_ receptor occupancy (RO) significantly increased reward responsiveness. This was revealed by significant increase in response bias (log *b*). **B** Response bias with low doses of amisulpride was significantly higher compared to vehicle. **C** Response bias with ENX-104 at low doses was significantly greater than vehicle. **B**, **C** Log *b* effect size comparison to respective vehicle controls revealed larger effect size of low dose ENX-104 (0.5 mg/kg: Cohen’s *d* ~ 1.2) as compared to amisulpride (1 mg/kg: Cohen’s *d* ~ 0.8) at corresponding low doses. Data are presented as means ± SEM. *p < 0.05, **p < 0.01, n = 8/group.

Impact of ENX-104 on reward responsiveness was quantitatively characterized in rats using the PRT (Fig. 4) [23, 25, 28, 31]. Prior to testing ENX-104, the assay was validated with amisulpride, which has been historically used at low doses for the treatment of dysthymia in patients[14, 19, 33, 34]. As predicted, following 13.5 (±1.5) sessions of training, PRT test sessions following administration of low doses of amisulpride (1 and 5 mg/kg) were characterized by enhancement of response bias (log *b*) as compared to vehicle. Specifically, analyses showed that 1 mg/kg was significantly different than vehicle (paired t-test analyses: *t*[7] = 2.20; p = 0.03; d = 0.79), as was 5 mg/kg of amisulpride (*t*[7] = 2.08; p = 0.04; d = 0.74) (Fig. 4B). Because low (1 and 5 mg/kg) but not higher doses (50 mg/kg) of amisulpride increased log *b*, general linear model tests with within-subjects contrast (quadratic term) were conducted, which yielded a trend towards statistical significance (*F*[1,7] = 4.80; *p* = 0.065).

Next, the impact of ENX-104 at three dose levels (0.5, 1, and 2.5 mg/kg) was examined in the rat PRT. Following 15.3 ( ± 0.6) sessions of training, PRT test sessions following administration of low dose levels, 0.5 and 1 mg/kg ENX-104 significantly increased reward responsiveness (log *b*) to 0.44 ± 0.11 and 0.48 ± 0.1, respectively as compared to vehicle treatment 0.27 ± 0.05 (Fig. 4C). Because the higher dose of ENX-104 (2.5 mg/kg) was not hypothesized to increase reward responsiveness, a one-way ANOVA was not an appropriate statistical analysis of dose-response function. Instead, general linear model tests with within-subjects contrast (quadratic term) were conducted and yielded statistical significance across the dose-response function (*F*[1,7] = 5.69; p = 0.048). Additionally, given the a priori hypotheses, paired t-test analyses between vehicle and ENX-104 dose groups enabled separate appraisal of statistical significance for dose-related effects. These analyses showed that 0.5 mg/kg was significantly greater than vehicle (*t*[7] = 3.4; p = 0.006; d = 1.206), as was 1 mg/kg ENX-104 (*t*[7] = 2.13; p = 0.04; d = 0.753), but not 2.5 mg.kg ENX-104 (*t*[7] = 1.41; p = 0.10; d = 0.498). It is important to note that this effect was revealed by substantive increases in log *b* without reductions in task discriminability (log *d*) for ENX-104 or amisulpride (Supplementary Fig. S2).

Data from a receptor occupancy study with amisulpride helped predict D_2_ receptor occupancy at different dose levels. Using the model derived from our empirical data (data not shown), we chose these three doses of amisulpride to help cover an receptor occupancy range from 20-70%, enabling a direct comparison of the behavioral effects of ENX-104 and amisulpride. Based on this, the 50 mg/kg dose corresponded to 55-75% D_2_ receptor occupancy range, in a range similar to the higher dose of ENX-104 (2.5 mg/kg). Cohen’s *d* values were computed to compare effect sizes across treatments. Effect sizes for ENX-104-treated groups at 0.5, 1, and 2.5 mg/kg versus the vehicle-treated group were 1.2, 0.75, and 0.5, respectively. Whereas Cohen’s *d* values for amisulpride-treated groups at 1, 5, and 50 mg/kg dose levels versus the vehicle-treated group were 0.79, 0.74, and 0.19, respectively (Fig. 4B, C). Notably, computation of Cohen’s *d* values across experiments revealed that the 0.5 mg/kg ENX-104 dose was linked to the largest effect across all dose levels tested (Supplementary Fig. S2) with an effect size greater than that of low dose amisulpride at equivalent estimated D_2_/D_3_ receptor occupancy (Fig. 4B, C). Highlighting specificity, ENX-104 did not affect discriminability (Supplementary Fig. S2), suggesting that changes in task difficulty did not confound the response bias findings.

### Integrated model of ENX-104 efficacy at various D_2_/D_3_ receptor occupancies

D_2_/D_3_ antagonists are known to display antipsychotic efficacy and higher doses are associated with motor side effects including extrapyramidal symptoms (EPS). Among the multiple assays available for testing antipsychotic efficacy, such as amphetamine induced hyperlocomotion (modeling psychotic symptoms) and prepulse inhibition (modeling sensorimotor deficits), we utilized the conditioned avoidance response (CAR) assay for broader sensitivity to antipsychotic efficacy [35]. ENX-104, specifically at higher doses tested (2.5 and 5 mg/kg), showed significant positive effects in the rat CAR (*F*[4,49] = 49.1; p < 0.001), with mean values of 66.2 ± 8.9% and 17.5 ± 3.61% avoidance response, respectively, compared to vehicle treated controls (98.4 ± 1.57%). In contrast, the lower dose of 0.5 mg/kg did not impact avoidance response in the CAR (84.6 ± 7.08%) as compared to controls (Supplementary Fig. S3). In addition, ENX-104 at dose levels of 0.5 and 2.5 mg/kg did not increase (0 escape failures at these dose levels) the number of escape failures which is associated with motor impairment. Only the higher dose level tested, 5 mg/kg, showed a significant increase in escape failures compared to vehicle (median of 2.5 escape failures as compared to 0 in vehicle treated controls) (Supplementary Fig. S3). Notably, ENX-104 at dose levels (0.5 mg/kg) associated with enhanced reward response did not show antipsychotic efficacy, nor motor impairment in the CAR assay.

The induction of catalepsy in rodents is an assay used for detecting antipsychotic drugs that induce extrapyramidal symptoms (EPS). The effects of ENX-104 at three dose levels (1, 2.5, and 10 mg/kg, po) on catalepsy were assessed at 4 h post-dose in rats. ENX-104, at 1 and 2.5 mg/kg dose levels (mean catalepsy of 0 out of 4 for each dose group), did not induce catalepsy compared to vehicle-treated controls (mean catalepsy score of 0). Only at the highest dose level of 10 mg/kg, ENX-104 induced catalepsy at 4 h post-dose (increased mean catalepsy score of 2.8 + 0.25 (p < 0.001) and latency for paw withdrawal 53.6 + 1.66 (p < 0.001) (Supplementary Fig. S4). Notably, no catalepsy was observed at dose levels associated with enhanced reward response (1 mg/kg) or antipsychotic like effects (2.5 mg/kg).

**Fig. 5:**
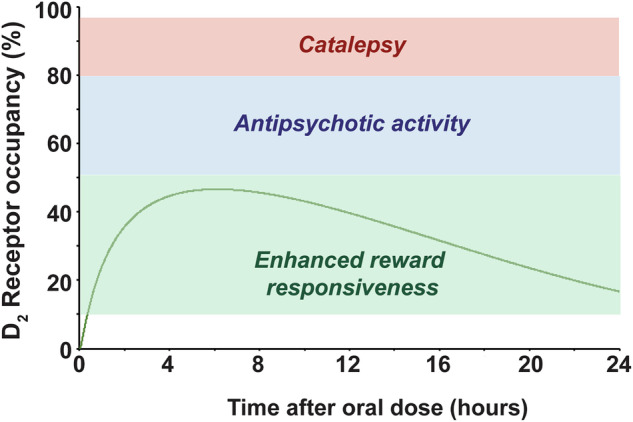
Integrated  model of ENX-104 efficacy. An integrated model of ENX-104 efficacy indicates that D_2_/D_3_ receptor occupancy of ~10–50% is the target range for enhancing reward responsiveness which may correlate with anti-anhedonic effects (green shade), and antipsychotic effects at 50-80% (blue shade) and catalepsy emerging only at receptor occupancy (RO) greater than ~80% (red shade). A model incorporating pharmacokinetics and pharmacodynamic effects of ENX-104 indicates that a single oral dose (1 mg/kg in rat) would produce sustained target engagement over 24 h within the target range for potential anti-anhedonic activity (green line).

By integrating ENX-104 brain exposure data from the PRT, CAR and catalepsy assays, it can be predicted that D_2_/D_3_ receptor occupancy of ~10–50% is associated with enhanced reward responsiveness, ~65-80% receptor occupancy is associated with antipsychotic activity and >80% receptor occupancy is associated with catalepsy (Fig. 5). Integrating data from brain ENX-104 levels, at the 1 mg/kg dose we predict sustained D_2_/D_3_ receptor occupancy in the reward-response enhancing receptor occupancy range over the course of 24 h (Fig. 5).

## Discussion

We hypothesized that a selective D_2_/D_3_ receptor antagonist, administered at low doses (corresponding to low D_2_/D_3_ receptor occupancy) would preferentially block presynaptic autoreceptors, increase dopamine in reward-related circuits and enhance reward responsiveness. In support of this, our microdialysis studies revealed that while the dopamine releasing agent amphetamine increased dopamine levels, co-administration with ENX-104 resulted in an even greater and more sustained elevation of striatal dopamine, consistent with enhanced dopamine release via blockade of feedback inhibition. Under physiological conditions, ENX-104 increased dopamine levels in the nucleus accumbens at doses of 1 mg/kg and 2.5 mg/kg, corresponding to approximately 40% and 70% D_2_/D_3_ RO, respectively. Interestingly, while both doses elevated dopamine, their behavioral effects in the PRT and CAR diverged.

In the rat PRT, a translational preclinical assay to objectively quantify enhancement of reward responsiveness [31], only low doses (0.5 and 1 mg/kg, ~10–50% RO) increased response bias for the richly rewarded stimulus. Consistent with our hypotheses, higher doses (2.5 mg/kg, ~65–80% RO) did not affect reward responsiveness in the rat PRT but, rather, showed antipsychotic efficacy in the conditioned avoidance response (CAR) test, a validated rodent model of antipsychotic activity [35]. As expected, 0.5 mg/kg dose showed no effect in the rat CAR, indicating functional specificity at different D_2_ receptor occupancy thresholds.

ENX-104 also demonstrated a favorable side effect profile preclinically. Catalepsy, a rodent marker for EPS often associated with high-dose D_2_ receptor antagonism, was only observed at the highest tested dose (10 mg/kg), but not at doses producing both increases in reward responsiveness and antipsychotic effects (1 mg/kg and 2.5 mg/kg, respectively). Based on these preclinical data, ENX-104 is anticipated to carry low risk of motor side effects at doses that enhance reward responsiveness, suggesting a potentially favorable therapeutic window. However, this would have to be verified empirically in clinical trials. PK profiling in rats demonstrated rapid plasma clearance alongside robust brain enrichment, supporting the rationale for a once-daily dosing regimen. This PK profile underscores the compound’s potential safety and tolerability, though confirmation will require clinical evaluation. Importantly, all experiments in the present study were conducted in rodents, therefore, clinical translation will necessitate single- and multiple-ascending-dose PK studies combined with D_2_/D_3_ receptor PET imaging to establish safety and target engagement in humans. Although amisulpride is approved in several European countries for dysthymia, a chronic depressive condition marked by persistent low mood [18], there remains an unmet need for a selective and brain penetrant molecule for the treatment of reward response deficits.

ENX-104, as a brain-penetrant, highly selective D_2_/D_3_ receptor antagonist offers a promising new modality for treating reward-related deficits in MDD at low doses, meeting a critical need in depression therapeutics. As reviewed above, anhedonia is a complex heterogeneous construct. As such, deficits in reward responsiveness may reflect not only impaired hedonic capacity but also broader motivational or cognitive deficits. This distinction is particularly important in considering the role of dopamine, which primarily supports motivational processes such as incentive salience and reward learning, rather than the hedonic experience, per se [8, 11]. This nuance has further implications for translational research. In schizophrenia, for example, anhedonia (diminished experience of pleasure) can be dissociated from avolition (diminished goal-directed behavior) [36], while in depression the two often overlap [37]. Accordingly, animal models that show blunted responsiveness to reinforcers may capture reward learning abnormalities rather than subjective hedonic states. With the above caveats in mind, the PRT has nonetheless emerged as a useful measure of reward responsiveness, a behavioral phenotype associated with anhedonia. Thus, reduced response bias in MDD, specifically among MDD individuals with elevated anhedonic symptoms or the melancholic subtype of MDD [6, 23–25, 38–40], has been found to correlate with current [22, 41–43] and predict future [22, 25] anhedonic symptoms. Critically, and consistent with the notion that the PRT probes specific subdomains of reward processing (reward responsiveness and reward learning) rather than the full spectrum of anhedonic phenotypes, response bias has been found to explain 11-15% of the variance of anhedonic symptoms [22, 41, 43]. Nevertheless, in the context of drug development, the PRT offers a valuable translational tool for assessing whether pharmacological agents, such as compounds that modulate dopaminergic transmission, can enhance reward responsiveness, and thereby target a critical component of anhedonia.

## Supplementary information

Supplemental material

## Data Availability

Raw or processed data from the manuscript are available upon request.

## References

[1] Pizzagalli DA. Toward a better understanding of the mechanisms and pathophysiology of anhedonia: are we ready for translation?. Am J Psychiatry. 2022;179:458–69. 10.1176/appi.ajp.20220423. 35775159 10.1176/appi.ajp.20220423 PMC9308971

[2] Kelly CA, Freeman KB, Schumacher JA. Treatment-resistant depression with anhedonia: Integrating clinical and preclinical approaches to investigate distinct phenotypes. Neurosci Biobehav Rev. 2022;136:104578. 10.1016/j.neubiorev.2022.104578. 35176319 10.1016/j.neubiorev.2022.104578

[3] Mizuno Y, Ashok AH, Bhat BB, Jauhar S, Howes OD. Dopamine in major depressive disorder: A systematic review and meta-analysis of in vivo imaging studies. J Psychopharmacol. 2023;37:1058–69. 10.1177/02698811231200881. 37811803 10.1177/02698811231200881 PMC10647912

[4] Peciña M, Sikora M, Avery ET, Heffernan J, Peciña S, Mickey BJ, et al. Striatal dopamine D2/3 receptor-mediated neurotransmission in major depression: Implications for anhedonia, anxiety and treatment response. Eur Neuropsychopharmacol. 2017;27:977–86. 10.1016/j.euroneuro.2017.08.427. 28870407 10.1016/j.euroneuro.2017.08.427 PMC5623119

[5] Hasler G, Fromm S, Carlson PJ, Luckenbaugh DA, Waldeck T, Geraci M, et al. Neural response to catecholamine depletion in unmedicated subjects with major depressive disorder in remission and healthy subjects. Arch Gen Psychiatry. 2008;65:521–31. 10.1001/archpsyc.65.5.521. 18458204 10.1001/archpsyc.65.5.521 PMC2676777

[6] Whitton AE, Reinen JM, Slifstein M, Ang YS, McGrath PJ, Iosifescu DV, et al. Baseline reward processing and ventrostriatal dopamine function are associated with pramipexole response in depression. Brain. 2020;143:701–10. 10.1093/brain/awaa002. 32040562 10.1093/brain/awaa002 PMC7009463

[7] Rizvi SJ, Pizzagalli DA, Sproule BA, Kennedy SH. Assessing anhedonia in depression: Potentials and pitfalls. Neurosci Biobehav Rev. 2016;65:21–35. 10.1016/j.neubiorev.2016.03.004. 26959336 10.1016/j.neubiorev.2016.03.004 PMC4856554

[8] Webber HE, Lopez-Gamundi P, Stamatovich SN, de Wit H, Wardle MC. Using pharmacological manipulations to study the role of dopamine in human reward functioning: A review of studies in healthy adults. Neurosci Biobehav Rev. 2021;120:123–58. 10.1016/j.neubiorev.2020.11.004. 33202256 10.1016/j.neubiorev.2020.11.004 PMC7855845

[9] Berridge KC, Kringelbach ML. Pleasure systems in the brain. Neuron. 2015;86:646–64. 10.1016/j.neuron.2015.02.018. 25950633 10.1016/j.neuron.2015.02.018 PMC4425246

[10] Alcaro A, Huber R, Panksepp J. Behavioral functions of the mesolimbic dopaminergic system: an affective neuroethological perspective. Brain Res Rev. 2007;56:283–321. 10.1016/j.brainresrev.2007.07.014. 17905440 10.1016/j.brainresrev.2007.07.014 PMC2238694

[11] Berridge KC, Kringelbach ML. Affective neuroscience of pleasure: reward in humans and animals. Psychopharmacol (Berl). 2008;199:457–80. 10.1007/s00213-008-1099-6. 10.1007/s00213-008-1099-6 PMC3004012 18311558

[12] Richard JM, Castro DC, Difeliceantonio AG, Robinson MJ, Berridge KC. Mapping brain circuits of reward and motivation: in the footsteps of Ann Kelley. Neurosci Biobehav Rev. 2013;37:1919–31. 10.1016/j.neubiorev.2012.12.008. 23261404 10.1016/j.neubiorev.2012.12.008 PMC3706488

[13] Lindgren N, Usiello A, Goiny M, Haycock J, Erbs E, Greengard P, et al. Distinct roles of dopamine D2L and D2S receptor isoforms in the regulation of protein phosphorylation at presynaptic and postsynaptic sites. Proc Natl Acad Sci USA. 2003;100:4305–9. 10.1073/pnas.0730708100. 12651945 10.1073/pnas.0730708100 PMC153088

[14] Boyer P, Lecrubier Y, Stalla-Bourdillon A, Fleurot O. Amisulpride versus amineptine and placebo for the treatment of dysthymia. Neuropsychobiology. 1999;39:25–32. 10.1159/000026556. 9892856 10.1159/000026556

[15] Papp M, Wieronska J. Antidepressant-like activity of amisulpride in two animal models of depression. J Psychopharmacol. 2000;14:46–52. 10.1177/026988110001400106. 10757253 10.1177/026988110001400106

[16] Rittmannsberger H. Amisulpride as an augmentation agent in treatment resistant depression: a case series and review of the literature. Psychiatr Danub. 2019;31:148–56. 10.24869/psyd.2019.148. 31291218 10.24869/psyd.2019.148

[17] Admon R, Kaiser RH, Dillon DG, Beltzer M, Goer F, Olson DP, et al. Dopaminergic enhancement of striatal response to reward in major depression. Am J Psychiatry. 2017;174:378–86. 10.1176/appi.ajp.2016.16010111. 27771973 10.1176/appi.ajp.2016.16010111 PMC5378658

[18] Zangani C, Giordano B, Stein HC, Bonora S, D'Agostino A, Ostinelli EG. Efficacy of amisulpride for depressive symptoms in individuals with mental disorders: a systematic review and meta-analysis. Hum Psychopharmacol. 2021;36:e2801. 10.1002/hup.2801. 34727399 10.1002/hup.2801 PMC8596405

[19] Lecrubier Y, Boyer P, Turjanski S, Rein W. Amisulpride versus imipramine and placebo in dysthymia and major depression. Amisulpride Study Group J Affect Disord. 1997;43:95–103. 10.1016/s0165-0327(96)00103-6. 10.1016/s0165-0327(96)00103-6 9165379

[20] Kondo T, Mihara K, Yasui N, Nagashima U, Ono S, Kaneko S, et al. Therapeutic spectrum of nemonapride and its relationship with plasma concentrations of the drug and prolactin. J Clin Psychopharmacol. 2000;20:404–9. 10.1097/00004714-200008000-00003. 10917400 10.1097/00004714-200008000-00003

[21] Satoh K, Someya T, Shibasaki M. Effects of nemonapride on positive and negative symptoms of schizophrenia. Int Clin Psychopharmacol. 1996;11:279–81. 10.1097/00004850-199612000-00010. 9031995 10.1097/00004850-199612000-00010

[22] Pizzagalli DA, Jahn AL, O’Shea JP. Toward an objective characterization of an anhedonic phenotype: a signal-detection approach. Biol Psychiatry. 2005;57:319–27. 10.1016/j.biopsych.2004.11.026. 15705346 10.1016/j.biopsych.2004.11.026 PMC2447922

[23] Fletcher K, Parker G, Paterson A, Fava M, Iosifescu D, Pizzagalli DA. Anhedonia in melancholic and non-melancholic depressive disorders. J Affect Disord. 2015;184:81–88. 10.1016/j.jad.2015.05.028. 26074016 10.1016/j.jad.2015.05.028 PMC4519400

[24] Pizzagalli DA, Iosifescu D, Hallett LA, Ratner KG, Fava M. Reduced hedonic capacity in major depressive disorder: evidence from a probabilistic reward task. J Psychiatr Res. 2008;43:76–87. 10.1016/j.jpsychires.2008.03.001. 18433774 10.1016/j.jpsychires.2008.03.001 PMC2637997

[25] Vrieze E, Pizzagalli DA, Demyttenaere K, Hompes T, Sienaert P, de Boer P, et al. Reduced reward learning predicts outcome in major depressive disorder. Biol Psychiatry. 2013;73:639–45. 10.1016/j.biopsych.2012.10.014. 23228328 10.1016/j.biopsych.2012.10.014 PMC3602158

[26] Luc OT, Pizzagalli DA, Kangas BD. Toward a quantification of anhedonia: unified matching law and signal detection for clinical assessment and drug development. Perspect Behav Sci. 2021;44:517–40. 10.1007/s40614-021-00288-w. 35098023 10.1007/s40614-021-00288-w PMC8738811

[27] Pizzagalli DA, Goetz E, Ostacher M, Iosifescu DV, Perlis RH. Euthymic patients with bipolar disorder show decreased reward learning in a probabilistic reward task. Biol Psychiatry. 2008;64:162–8. 10.1016/j.biopsych.2007.12.001. 18242583 10.1016/j.biopsych.2007.12.001 PMC2464620

[28] Kangas BD, Wooldridge LM, Luc OT, Bergman J, Pizzagalli DA. Empirical validation of a touchscreen probabilistic reward task in rats. Transl Psychiatry. 2020;10:285. 10.1038/s41398-020-00969-1. 32792526 10.1038/s41398-020-00969-1 PMC7426406

[29] Adam AS, LaMalfa KS, Razavi Y, Kohut SJ, Kangas BD. A multimodal preclinical assessment of MDMA in female and male rats: prohedonic, cognition disruptive, and prosocial effects. Psychedelic Med. 2024;2:96–108. 10.1089/psymed.2023.0049. 10.1089/psymed.2023.0049 PMC11324000 39149579

[30] Jenkins AR, Radl DB, Kornecook TJ, Pizzagalli DA, Bergman J, Buhl DL, et al. Environmental determinants of ketamine’s prohedonic and antianhedonic efficacy: Persistence of enhanced reward responsiveness is modulated by chronic stress. J Pharm Exp Ther. 2025;392:103572. 10.1016/j.jpet.2025.103572. 10.1016/j.jpet.2025.103572 PMC13095460 40288209

[31] Kangas BD, Der-Avakian A, Pizzagalli DA. Probabilistic reinforcement learning and anhedonia. Curr Top Behav Neurosci. 2022;58:355–77. 10.1007/7854_2022_349. 35435644 10.1007/7854_2022_349 PMC11708784

[32] Kangas BD, Bergman J. Touchscreen technology in the study of cognition-related behavior. Behav Pharm. 2017;28:623–9. 10.1097/FBP.0000000000000356. 10.1097/FBP.0000000000000356 PMC5687822 29064843

[33] Ravizza L. Amisulpride in medium-term treatment of dysthymia: a six-month, double-blind safety study versus amitriptyline. AMILONG investigators. J Psychopharmacol. 1999;13:248–54. 10.1177/026988119901300307. 10512080 10.1177/026988119901300307

[34] Natesan S, Reckless GE, Barlow KB, Nobrega JN, Kapur S. Amisulpride the ‘atypical’ atypical antipsychotic-comparison to haloperidol, risperidone and clozapine. Schizophr Res. 2008;105:224–35. 10.1016/j.schres.2008.07.005. 18710798 10.1016/j.schres.2008.07.005

[35] Wadenberg ML, Hicks PB. The conditioned avoidance response test re-evaluated: is it a sensitive test for the detection of potentially atypical antipsychotics?. Neurosci Biobehav Rev. 1999;23:851–62. 10.1016/s0149-7634(99)00037-8. 10541060 10.1016/s0149-7634(99)00037-8

[36] Strauss GP, Horan WP, Kirkpatrick B, Fischer BA, Keller WR, Miski P, et al. Deconstructing negative symptoms of schizophrenia: avolition-apathy and diminished expression clusters predict clinical presentation and functional outcome. J Psychiatr Res. 2013;47:783–90. 10.1016/j.jpsychires.2013.01.015. 23453820 10.1016/j.jpsychires.2013.01.015 PMC3686506

[37] Treadway MT, Zald DH. Reconsidering anhedonia in depression: lessons from translational neuroscience. Neurosci Biobehav Rev. 2011;35:537–55. 10.1016/j.neubiorev.2010.06.006. 20603146 10.1016/j.neubiorev.2010.06.006 PMC3005986

[38] Liu WH, Chan RC, Wang LZ, Huang J, Cheung EF, Gong QY, et al. Deficits in sustaining reward responses in subsyndromal and syndromal major depression. Prog Neuropsychopharmacol Biol Psychiatry. 2011;35:1045–52. 10.1016/j.pnpbp.2011.02.018. 21371518 10.1016/j.pnpbp.2011.02.018

[39] Costi S, Morris LS, Collins A, Fernandez NF, Patel M, Xie H, et al. Peripheral immune cell reactivity and neural response to reward in patients with depression and anhedonia. Transl Psychiatry. 2021;11:565. 10.1038/s41398-021-01668-1. 34741019 10.1038/s41398-021-01668-1 PMC8571388

[40] Xiao J, Adkinson JA, Myers J, Allawala AB, Mathura RK, Pirtle V, et al. Beta activity in human anterior cingulate cortex mediates reward biases. Nat Commun. 2024;15:5528. 10.1038/s41467-024-49600-7. 39009561 10.1038/s41467-024-49600-7 PMC11250824

[41] Reilly EE, Whitton AE, Pizzagalli DA, Rutherford AV, Stein MB, Paulus MP, et al. Diagnostic and dimensional evaluation of implicit reward learning in social anxiety disorder and major depression. Depress Anxiety. 2020;37:1221–30. 10.1002/da.23081. 32906219 10.1002/da.23081

[42] Chevallier C, Tonge N, Safra L, Kahn D, Kohls G, Miller J, et al. Measuring social motivation using signal detection and reward responsiveness. PLoS One. 2016;11:e0167024 10.1371/journal.pone.0167024. 27907025 10.1371/journal.pone.0167024 PMC5132309

[43] Bogdan R, Pizzagalli DA. Acute stress reduces reward responsiveness: implications for depression. Biol Psychiatry. 2006;60:1147–54. 10.1016/j.biopsych.2006.03.037. 16806107 10.1016/j.biopsych.2006.03.037 PMC2288705

