# Supplementary material for: ENX-104: a selective and potent D2/D3 receptor antagonist enhances dopamine neurotransmission and reward responsiveness in translational rodent models
Source: Neuropsychopharmacology. 2025 Dec 7;51(6):1065–73. doi: 10.1038/s41386-025-02287-w (PMC13125307; doi:10.1038/s41386-025-02287-w)
Supplement: Supplementary file 1 — Supplemental material [file 41386_2025_2287_MOESM1_ESM.docx]

**SUPPLEMENTARY MATERIAL**

**SUPPLEMENTAL METHODS**

***In vitro pharmacology***

Binding and functional activity of ENX-104 at human dopaminergic and serotonergic receptors were tested using recombinant cell lines (Chinese Hamster Ovary; [CHO]-K1) expressing respective receptors. Binding assays were conducted using radiolabeled reference tracers for each receptor and compared against reference comparators. ENX-104 was tested at eight concentrations in duplicate to test for radioligand binding, agonist, or antagonist activity. Agonist activity is expressed as a percent of the activity of the reference agonist at its EC_100_ concentration. Antagonist activity of the test compound is expressed as a percentage of the inhibition of the reference agonist at its EC_80_ concentration. Data was averaged values derived from multiple experiments.

For determining binding potency and functional activity assays, radiolabeled tracers/reference comparators were used as listed below.

*Binding potency:*

| **Receptor** | **Cell line** | **Reference tracer** | **Reference competitor** |
| --- | --- | --- | --- |
| D2L | CHO-K1 | [^3^H]-Spiperone | Risperidone |
| D2S | CHO-K1 | [^3^H]-Spiperone | Risperidone |
| D3 | CHO-K1 | [^3^H]-R-(+)-7-OH-DPAT | R-(+)-7-OH-DPAT |
| D4.4 | CHO-K1 | [^3^H]-Spiperone | Haloperidol |
| human D2L | CHO-K1 | [^3^H]-Spiperone | Risperidone |

*Functional activity:*

| **Receptor** | **Assay** | **Cell line** | **Reference agonist** | **Reference antagonist** |
| --- | --- | --- | --- | --- |
| D2L | cAMP | CHO-K1 | Quinpirole | Haloperidol |
| D2S | cAMP | CHO-K1 | Quinpirole | Haloperidol |
| D3 | GTP | CHO-K1 | Dopamine | GR103691 |
| D4.4 | cAMP | CHO-K1 | Dopamine | Spiperone |

***Pharmacokinetic studies***

The PK and brain penetration of ENX-104 was determined following oral administration to male Sprague Dawley rats. Rat (n = 3 per dose group) received a single PO dose of 5 mg/kg of ENX-104 and serial blood samples were collected at 5, 10, and 30 minutes and at 1, 2, 4, and 8 hours post-dose. Terminal blood and brain tissue samples were collected at 24 hours post-dose. Two additional groups of animals (n = 3 per dose group / time point) received a single PO dose 5 mg/kg of ENX-104 and terminal blood and brain tissue were collected from three animals per time point in each dose group at 1, 4, and 8 hours post-dose. Plasma was obtained by centrifugation and brain tissue samples were homogenized in water. Concentrations of ENX-104 in plasma and brain were determined by LC-MS/MS. The PK parameters were determined using a NCA of Phoenix WinNonlin (v8.1) software. Plasma PK parameters for ENX-104 from the serial sampling (n = 3/timepoint).

*Plasma samples processing:*

Aliquots (30 µL) of each plasma sample were mixed with 30 µL of ACN: water [50:50, v/v] (diluent) and then extracted using 200 µL of IS (200 ng/mL verapamil) solution in ACN. The samples were vortexed vigorously for 10 minutes and then centrifuge at 4000 rpm for 10 minutes at 15°C. After centrifugation, supernatant aliquots of 100 µL were diluted with water (100 µL) and aliquots of 5 µL were injected for LC‑MS/MS analysis.

*Brain tissue processing:*

The pre-weighed brain tissue samples were transferred to separate polypropylene tubes and three volumes of water and one ceramic bead were added for homogenization. The samples were homogenized with a FastPrep-24™ 5G Tissue and Cell Homogenizer (MP Biomedical, LLC, Santa Ana, CA) set to a speed of 4.0 m/sec for 20 sec × 3. An aliquot (15 µL) of each brain tissue sample homogenate was mixed with 15 µL of control rat plasma to yield samples with a total dilution of 8. The diluted samples were mixed with 30 µL of diluent and then extracted using 200 µL of IS solution. The samples were then processed as described above.

Aliquots of each working stock were serially diluted with diluent to yield separate calibration standard solutions at concentrations of 20.0, 10.0, 5.00, 2.00, 1.00, 0.500, 0.200, 0.100, 0.0500, 0.0200 and 0.0100 ng/mL for ENX-104. Aliquots (30 μL) of each calibration standard solution were mixed with 30 μL of control rat plasma and then extracted using 200 μL of IS solution. The standards were then processed as described above.

Concentrations of ENX-104 in quality control, plasma and brain tissue samples were determined by LC‑MS/MS analysis using verapamil as the IS. Separations were performed with a Shimadzu LC pump and autosampler (Shimadzu Scientific Instruments, Columbia, MD) using an ACE 5 Excel C18, 2.1 × 50 mm, 5 µm column (Advanced Chromatography Technologies Ltd, Aberdeen, Scotland) maintained at ambient temperature. The mobile phase consisted of 0.1% aqueous formic acid (eluent A) and 0.1% formic acid in ACN (eluent B). A SCIEX Triple Quad^™^ 6500+ LC‑MS/MS system (SCIEX, Framingham, MA) equipped with an electrospray ionization (ESI) source was used as the detector. The instrument was operated in positive ion mode using multiple reaction monitoring (MRM) with specific precursor-product ion pairs for ENX-104 and verapamil.

Non-compartmental analyses were performed to calculate the pharmacokinetics of ENX-104 in male SD rats. The maximum plasma concentration (C_max_) and time to maximum plasma concentration (t_max_) were obtained directly from the measured plasma concentrations of ENX-104. The area under the concentration-time curve (AUC) was calculated from zero to the last quantifiable concentration using the linear trapezoidal method. Phoenix® WinNonlin® (version 8.4) (Certara USA, Inc., Princeton, NJ) was used to generate pharmacokinetic data.

***Receptor occupancy studies***

Sprague-Dawley rats from Charles River (Margate) were obtained and group housed (2 or 3 to a cage) at an ambient temperature of 21±2°C on a normal 12-hour light/dark cycle (lights on 07:00). Relative humidity was typically 55±15% with prolonged periods below 40% RH (Relative Humidity) avoided as detailed in the UK Code of Practice. As a refinement, each cage contained a red plastic tunnel and chew stick. Standard pelleted diet and filtered water were available ad libitum. Upon arrival, rats were weighed and given wet mash overnight (standard maintenance diet mixed with water) to aid recovery from transport.

Animals were allocated to treatment groups based on body weight and cage so animals in the same cage received the same treatment. On the day of testing, animals were dosed orally with either vehicle (0.5% methylcellulose) or ENX-104 at a single dose (2.5 mg/kg) or the positive control olanzapine (10 mg/kg) (n = 5 / group). Rats were humanely killed by a Schedule 1 method (increasing exposure to CO2 with confirmation of death by cervical dislocation) at 1, 2, 4, 8 or 24 hours after ENX-104 administration or 1 hour after vehicle or olanzapine administration.

A post-mortem blood sample (~ 5 ml) was taken by cardiac puncture and placed into K/EDTA tubes (32.332, Sarstedt). The post-mortem blood samples were gently inverted, centrifuged (1900 g for 5 minutes at 4°C) and 1 ml of plasma from each animal was placed into a screwcap microtube (CP5915, Alpha Laboratories) for PK determination. All plasma samples were frozen and stored at -80°C.

Whole brains were removed, rinsed with saline and blot dried. The left striatum (~35-50 mg) and right striatum (approx. 35-50 mg) were dissected out and weighed before being frozen on dry ice. The striata from each hemisphere were frozen separately. The tissue was wrapped in aluminum placed in bags and stored at -20°C until the day of the appropriate study’s binding assay. The remaining brain tissue was weighed, frozen on dry ice, then wrapped in aluminum foil, placed in bags and stored at -80°C prior to shipping along with the plasma samples for bioanalysis.

The striata were homogenized individually in ice-cold 50 mM Tris, pH 7.4, 120 mM NaCl, 5 mM KCl, 2 mM CaCl2, 1 mM MgCl2 and 10 μM pargyline using a tight-fitting glass/Teflon homogeniser equivalent to 6.25 mg wet weight of tissue/ml and used immediately in the binding assay. Striatal homogenates (400 μl, equivalent to 2.5 mg wet weight tissue/tube) were incubated with 50 μl of 1.6 nM [^3^H]raclopride and either 50 μl assay buffer (total binding) or 50 μl of 1 μM (-)Sulpiride (to define non-specific binding) for 30 minutes at 23°C. The assay buffer consisted of 50 mM Tris, pH 7.4, 120 mM NaCl, 5 mM KCl, 2 mM CaCl2, 1 mM MgCl2 and 10 μM pargyline. The was buffer consisted of 50 mM Tris, pH 7.4. There were two tubes for the determination of total binding and two tubes for the determination of non-specific binding. Membrane bound radioactivity was recovered by filtration under vacuum through Skatron 11731 filters, pre-soaked in 0.5% polyethyleneimine (PEI) using a Skatron cell harvester. Filters were rapidly washed with ice-cold buffer (was setting 9, 9, 0) and radioactivity was determined by liquid scintillation counting (1 ml Packard MV Gold scintillator).

The value for specific binding in disintegrations per minute (DPM) was generated by subtraction of mean non-specific binding (DPM) from mean total binding (DPM) for each animal. Data was presented as mean specific binding (DPM), mean specific binding as a percentage of the vehicle-treated control taken as 100% and as mean receptor occupancy as a percentage of control taken as 0%. All data was square root transformed and analyzed by one-way ANOVA. ENX-104 was compared to vehicle by Dunnett’s test. Olanzapine was compared to vehicle by multiple t-test. Means were back transformed and adjusted for difference between vehicle and olanzapine groups between studies. P value < 0.05 was considered significant.

***In vivo microdialysis***

Experiments were carried out in male Sprague Dawley rats (~26-384 g in weight or approximately 8-10 weeks old at the time of the experiment; Charles River, UK). Animals were housed in groups of three (PK study) and four (microdialysis study) on a 12 h/12 h light/dark cycle (lights on at 07.00 h), at an average ambient temperature of 19.96 23.04°C. Average relative humidity was 40.42 66.92%. Standard pelleted rat diet (Teklad Certified Global 18% Protein Rodent Diet; Envigo) and filtered tap water were available ad libitum. Rats were weighed on the day of their arrival and provided with wet mash overnight to negate any weight loss during transportation, in addition to their diet pellets. The wet mash consisted of their standard rodent diet in powdered form mixed with water. They were weighed again the day after arrival and the mash was removed. Animals were allowed to acclimatize to these conditions for at least 1 week and were weighed/handled for three days prior to use (excluding weekends). These experiments were performed in strict accordance with Home Office Guidelines and licensed under the Animals (Scientific Procedures) Act 1986 (Project Licence P58B4DA70).

For the surgery, rats were anaesthetised with isoflurane (5% to induce, 2% to maintain) in O_2_ (1 litre/min) delivered via an anaesthetic unit (Burtons Medical Equipment Ltd, UK). A dual-probe study was conducted whereby each rat had two concentric microdialysis probes (CMA 12 Elite probes, CMA Sweden) stereotaxically implanted into the prefrontal cortex (co‑ordinates: AP+3.2 mm; ML+/‑2.5 mm relative to bregma; DV-4.0 mm relative to the skull surface, 2 mm tip) and nucleus accumbens (co‑ordinates: AP+2.2 mm; ML+/‑1.5 mm relative to bregma; DV-8.0 mm relative to the skull surface, 2 mm tip). Co-ordinates were taken from Paxinos and Watson atlas. The upper incisor bar was set at 3.3 mm below the interaural line so that the skull surface between bregma and lambda was horizontal. Additional burr holes were made for skull screws (stainless steel) and the probes were secured using dental cement. Carprofen (Rimadyl, Zoetis) was administered for pain relief at least 30 min prior to animals regaining consciousness following surgery (5 mg/kg s.c.). Following surgery, animals were individually housed in microdialysis round-bottom bowls (245 mm internal diameter at base of bowl, 360 mm wall height, BASi) with the microdialysis probes connected to liquid swivels and a counter‑balanced arm to allow unrestricted movement. Rats were allowed a recovery period of at least 16 h with food and water available *ad libitum*. During this time, the probes were continuously perfused at a flow rate of 1.2 µl/min with an artificial cerebrospinal fluid (aCSF; Harvard Apparatus, UK) of the following electrolyte composition (in mM): sodium 150; potassium 3.0; magnesium 0.8; calcium 1.4; phosphate 1.0; chloride 155.0. Microdialysis experiments were performed the day after surgery with 8 rats per experimental session. Microdialysate samples were collected from freely-moving rats at 30 min intervals for a baseline period of 120 min prior to the administration of vehicle or drug. Samples were then collected for a further 8 hours (20 samples in total) after drug treatment at intvervals of 30 minutes (n =8/group). Throughout the experiment, probes were perfused at a flow rate of 1.2 µl/min. Samples (36 µl) were collected into Eppendorf vials (300 µl volume) containing 7.5 µl of preservative to prevent oxidation of the monoamines (thus total sample volume was 43.5 µl). Samples were frozen in dry ice immediately after collection and after the completion of each microdialysis experiment all samples were stored at ‑80ºC until analysis.

At the end of the experiments, rats were killed by a UK Home Office approved Schedule 1 method (Euthatal [pentobarbital sodium] overdose IP) and their brains rapidly removed and fixed in a 10% v/v formalin saline solution for a minimum of 5 days. If data were considered statistical outliers, the actual location of the probe was verified by comparing the tract of the probe against the desired position, using a stereotaxic atlas. If probes were found to be incorrectly located, the data from the rat in question was discarded from the study.

*Drugs*

Each rat was administered vehicle or drug by the oral (p.o.) route (via gavage) immediately followed by vehicle or drug via the intraperitoneal (IP) route. The oral vehicle was 0.5% methylcellulose (400 cP, Sigma‑Aldrich, Lot SLCB9094, pH 7.1) and a dose volume of 5 ml/kg p.o. was used. The IP vehicle was 0.9% saline (pH 5.6) and a dose volume of 2 ml/kg IP was used. All drug solutions were prepared on the day of use.

ENX‑104 was stored at ambient temperature and weighed into glass vials (Group E/F) or glass Duran bottles. Vehicle was added to the receptacles, which were then sonicated (15‑20 min) forming uniform suspensions suitable for p.o. administration. The suspensions were protected from light and stirred for 15 min prior to and throughout the dosing period. This resulted in solutions which were suitable for p.o. administration.

d‑Amphetamine sulfate (Tocris, Batch No. 7A/222903) was weighed into glass Duran bottles and vehicle was added. The bottles were briefly shaken (few sec) resulting in clear solutions suitable for IP dosing (pH 6.1). The solutions were shaken well prior to administration. A correction factor of 1.36 was used to correct from salt to base and the dose stated is for the free base.

All reagents used in high-performance liquid chromatography (HPLC) analysis were of HPLC grade. Phosphoric acid, EDTA and methanol were obtained from Fisher Scientific (UK). 1‑Octane sulphonic acid was purchased from Sigma Aldrich (UK). Dopamine hydrochloride (DA; Batch No. BCCH5705) and serotonin creatinine sulfate monohydrate (5-HT; Batch No. BCCD2035) were purchased from Sigma‑Aldrich (UK). Neutral buffered formalin (10%) was obtained in 20 ml CellStor™ pots from CellPath Ltd (UK). All solvents used for LC‑MS were LCMS grade from Fisher Scientific except the water which was deionised and filtered before use.

*HPLC*

Detection and subsequent quantification of DA in the microdialysis samples was based on reversed-phase, ion-pair HPLC coupled with electrochemical detection and involved the use of an ALEXYS™ monoamine analyser (Antec Scientific, The Netherlands). The system consisted of two separate analytical columns with precolumn filters that shared a dual loop autosampler allowing one sample to be analysed simultaneously by two systems optimised for different neurotransmitters. Two solvent delivery pumps (LC 110) were used to circulate the respective mobile phases and an Antec in line degassing unit was used to remove air. Samples (10 µl total) were injected onto the columns via an autosampler (AS 110) with a cooling tray set at 4 ºC. Antec DECADE II™ electrochemical detectors were used and Antec micro VT 03 cells employing a high density, glassy carbon working electrode combined with a salt bridge reference electrode. The electrode signal was integrated using Antec’s CLARITY™ data acquisition system. Individual standard stock solutions of DA (10.0 mM) were prepared by dissolution in a mixture of equal quantities of deionised water and preservative (in order to prevent oxidation) and stored at 4 ºC. A working solution of the standard solution containing neurotransmitters was prepared daily by dilution in aCSF.

*Data analysis*

Microdialysis data were log transformed. Baseline was defined as the geometric mean of the four pre-treatment samples (i.e. those collected at ‑90 min, -60 min, -30 min and 0 min). Data were log transformed and analysis was by robust regression using M estimation, Huber weighting, using the default parameter c=1.345 with treatment as a factor and log(baseline) as a covariate. Each time was analysed separately, together with means during each of the eight hours after dosing, 0‑2 and 0‑4 hours and the overall 0-8 hours after dosing. For calculation of hourly and 8 hourly means, missing data were imputed to be the geometric mean of the previous and subsequent values (if the 450-480 min value was missing, it was imputed to be equal to the 420-450 min value).

Comparisons to vehicle were by Williams’ test for ENX-104 and by the multiple t test for d‑amphetamine, and ENX‑104 + d-amphetamine. Comparisons to d‑amphetamine alone and to ENX-104 alone for ENX-104 + d-amphetamine were by the multiple t test. A p value of < 0.05 was considered statistically significant. One animal was excluded from the anlaysis for the nucleus accumbens due to poor chromatography.

***Probabilistic Reward Task***

Eight adult male Sprague Dawley rats obtained from Charles River Laboratories (Wilmington, MA) weighing between 250 and 300 grams were used in the present study. Animals were housed in a climate-controlled vivarium with a 12-h light/dark cycle (lights on at 7am). Animals were maintained at approximately 80% of their free-feeding weight via post-session portions of rodent chow and had unrestricted access to water in their home cage. Experimental sessions were conducted 5 days a week (Mon-Fri). The protocol for the present studies was approved by the Institutional Animal Care and Use Committee at McLean Hospital and in accordance with guidelines provided by the Committee on Care and Use of Laboratory Animals of the Institute of Laboratory Animals Resources, Commission on Life Sciences (National Research Council, 2011).

Details of the apparatus can be found in Kangas and Bergman (2017). Briefly, a custom-built Plexiglas chamber (25x30x35 cm) was situated in a sound- and light-attenuating enclosure (40x60x45 cm). A 17” touch-sensitive screen (1739L, ELO TouchSystems, Menlo Park, CA) comprised the inside right-hand wall of the enclosure. An infusion pump (PHM-100-5, Med Associates, St. Albans, VT) outside the enclosure was used to deliver sweetened condensed milk solution into the shallow reservoir of a custom-designed aluminum receptacle. The receptacle was mounted 3 cm above the floor bars and centered on the left-hand inside wall. Both touchscreen and fluid reservoir were easily accessible to the subject. A speaker bar (NQ576AT, Hewlett-Packard, Palo Alto, CA) mounted above the touchscreen was used to emit audible feedback. All experimental events and data collection were programmed in E-Prime Professional 2.0 (Psychology Software Tools, Inc., Sharpsburg, PA).

*Initial training*

Modified response-shaping techniques were used to train rats to engage with the touchscreen26. A 5x5 cm blue square on a black background was presented in different sections of the touchscreen (left, right, or center), with the proviso that its lower edge always was 10 cm above the floor bars. This required the rat to rear on its hind legs to reach the screen and make a touchscreen response with its paw. Each response was reinforced with 0.1 mL of 30% sweetened condensed milk and the delivery was paired with an 880 ms yellow screen flash and 440 Hz tone and followed by a 5-sec intertrial interval (ITI) blackout period. After responses were reliably observed with latencies <5 sec following stimulus presentation, line-length discrimination training commenced.

*Line-length discrimination training*

Discrete trials began with concurrent presentation of a white line presented 5 cm above left and right response boxes. The width of the line was always 7 cm, but the length of the line was either 30 cm or 15 cm and varied in a quasi-random fashion across 100-trial sessions (50 trials of each length). Subjects learned to respond to the left or right response box depending on the length of the white line (i.e., long line = respond left, short line = respond right, or vice versa).

Response box designation was counter-balanced across subjects. A correct response was reinforced as described above and was followed by a 5 sec ITI, whereas an incorrect response immediately resulted in a 5 sec ITI. A correction procedure27 was implemented during initial discrimination training—each incorrect trial was repeated until a correct response was made—and was discontinued after session-wide trial repeats were <5 in each trial type. Discrimination sessions continued without correction until accuracies for both line lengths were >75% correct for 3 consecutive sessions.

*Probabilistic Reward Task*

Following line-length discrimination training, probabilistic reinforcement schedules were introduced. Based on the human task protocol, a 3:1 rich/lean probabilistic schedule was arranged such that 60% of correct responses to one of the line lengths (e.g., long line = rich alternative) and 20% of correct responses to the other line length (e.g., short line = lean alternative) were rewarded. rich/lean line assignment was counterbalanced across subjects and 50 trials of each trial type were presented in a quasi-random sequence. These probabilistic contingencies were assessed across 5 consecutive sessions prior to initiation of drug testing.

*PRT Drug Tests*. Following the establishment of probabilistic contingencies, an acute drug testing protocol was arranged that included intermittent maintenance sessions in which correct responses on all trials were reinforced, control sessions in which 3:1 (60%:20%) rich/lean probabilistic contingencies were arranged and, no more than once per week, a drug testing session in which vehicle or a dose of ENV-104 (0.5, 1, or 2.5 mg/kg) was tested by administering it per os (p.o.) 4 hours prior to a 3:1 (60%:20%) probabilistic session. Doses of ENX-104 were tested in a mixed order across subjects using a Latin Square design. Vehicle (0.5% methylcellulose) and all doses of ENX-104 were tested in all 8 animals (n = 8 / group).

*Data Analysis*

The implementation of probabilistic contingencies yields two primary dependent measures: response bias and task discriminability. These can be quantified by examining the number of Correct and incorrect responses in rich and lean trial types using, respectively, log *b* and log *d* equations derived from signal detection theory.


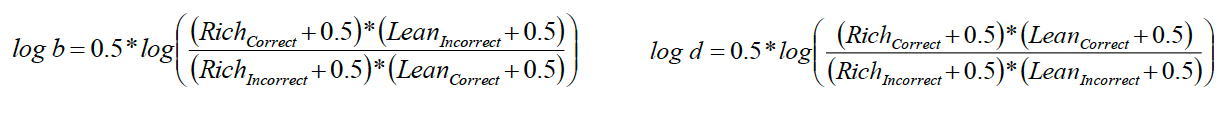


High bias values are produced by high numbers of correct responses during rich trials and incorrect responses during lean trials, which increase the log *b* numerator. High discriminability values are produced by high numbers of correct responses during both rich and lean trials, which increase the log *d* numerator. (0.5 is added to all parameters to avoid instances where no errors are made on a given trial type, which would make log transformation impossible.) Given the *a priori* hypotheses discussed above of small doses of ENX-104 increasing reward responsiveness (putatively due to presynaptic autoreceptor blockade) but high doses decreasing reward responsiveness (putatively due to postsynaptic blockade), log *b* data were subjected to general linear model tests of within-subjects contrast in which the quadradic term was specifically evaluated (an inverted U-function was expected). All other data (log *d*, accuracy, reaction time) were subject to repeated measures analysis of variance (ANOVA). For accuracy and reaction time, the repeated measures factor trial type (rich vs. lean) was added to the model. When appropriate, ANOVAs were followed by post-hoc tests (one-way: Dunnett’s multiple comparisons test; two-way: Bonferroni multiple comparisons test) to evaluate the statistical significance of stimulus type and drug treatment. Given *a priori* hypotheses (see below), paired t-tests and Cohen’s *d* were also used to evaluate, respectively, the statistical significance of differences in log *b* values and effect sizes following vehicle treatment and each individual dose of ENX-104. The criterion for significance was set at p<0.05. Effect sizes (Cohen’s *d* values) were interpreted using established conventions31: small (*d*=0.20), medium (*d*=0.50), and large effect (*d*>0.80). All statistical analyses were conducted using GraphPad Prism 9 Software (San Diego, CA, USA).

***Conditioned avoidance response***

Adult, male Wistar rats from Envigo (Indianapolis, IN) were used in this study. Rats were received at approximately 100‐150g, assigned unique identification numbers and group housed 2‐3 per cage in ventilated cages. Animals were maintained in a 12/12‐hour light/dark cycle with room temperature maintained at 22 ± 1°C and with the relative humidity maintained at approximately 50%. Food and water were provided ad libitum. All rats were examined, handled, and weighed prior to initiation of the study to assure adequate health and to minimize the non‐specific stress associated with testing. Each animal was randomly assigned across the treatment groups. The experiments were conducted during the animal’s light cycle phase. ENX‐104 (0.5, 2.5 and 5 mg/kg) was formulated in 0.5% Methyl Cellulose in water and administered orally at a dose volume of 1 ml/kg 4 hours prior to test.

The Conditioned Avoidance Response (CAR) Test has been shown to be a very reliable animal model for screening antipsychotic drugs. In the CAR paradigm, an animal is trained to respond to a conditioned stimulus (auditory and visual) by negative reinforcement (foot shock). Numerous studies have shown that typical and atypical antipsychotic drugs selectively suppress avoidance response in CAR, thus making it one the preferred assay to screen potential antipsychotic compounds. The CAR apparatus consists of a two‐way shuttle box with infra‐red (I/R) detection housed in sound‐attenuating chambers (Med Associates). The two‐way shuttle boxes have stainless steel grid floors and are partitioned by a guillotine door. Programs are run through Med‐PC version IV software. Rats were trained to avoid a foot shock following presentation of a light or tone. Rats were placed in the CAR two‐compartment shuttle box (Med Associates) and presented with a conditioned stimulus (CS; light and tone), followed by an aversive unconditioned stimulus (US;foot‐shock of 0.65 mA). Each rat goes through 20 trials with a variable ITI (20 – 60 sec). After several weeks of training in the CAR chambers, rats that passed the testing criterion of performing 16 ‐20 avoidance responses for three days in a row are included in the study and testing commenced. CAR testing consists of the same procedure as CAR training. Baseline was measured over three days prior to drug testing. Following the test, the rats were given one week washout between tests. The measures obtained from this test are:

Avoidance response: If the rat moved from one compartment to the other during the cuedstimulus (CS) presentation and prior to foot‐shock delivery. Decreased avoidance responding is the typical signature of an efficacious dose of an antipsychotic.

Escape Failure: If the rat failed to move into the other compartment during the 20‐sec footshock.

Avoidance response data were expressed as the number of avoidance responses as well as percent of avoidance responses based on the three baseline responses prior to drug test. Escape failures were expressed as the total number of failures during the test session. Dunnett’s post‐hoc comparisons when appropriate. Escape failures were analyzed by Kruskal‐Wallis nonparametric analysis followed by Dunnett’s post‐hoc comparisons when appropriate. Results are reported as mean ± SEM. An effect was considered significant if p<0.05 (n = 10 / group).

***Catalepsy***

Sprague-Dawley male rats (~161- 210 g body weight on arrival) were obtained from Charles River (Margate). Rats were group housed (2-4 animals/cage) on a 12 h/12 h light/dark cycle (lights on at 07.00h) at an ambient temperature of 21±3°C. Relative humidity was typically 55±15% with prolonged periods below 40% RH or above 70% RH avoided as detailed in the UK Code of Practice. As a refinement, each cage contained a red plastic tunnel and a chew stick. Teklad 2018C pelleted diet and filtered water was available *ad libitum*.

*Drugs*

Haloperidol (Product Code 0931; Lot: 4B/263582) was purchased from Tocris, Abingdon, UK. Haloperidol was prepared in saline and dosed intraperitoneally as a clear solution. ENX104 was prepared in 0.5% methylcellulose and dosed orally as a uniform fine suspension and all ENX-104 dosing preparations were stirred on a magnetic plate prior to and throughout dosing and protected from light. All compounds were formulated on the day of dosing and administered using a dose volume of 5 ml/kg. Drug doses are expressed as free base.

On the day of the test, animals were dosed with vehicle (po), ENX-104 (1, 2.5 and 10 mg/kg, po) or haloperidol (0.82 mg/kg, ip; ED_75_ dose). Animals were tested individually for catalepsy at 90, 180 (data not shown) and 240 minutes post-dose by gently placing each paw in turn on a large rubber bung (42 mm high, 45 mm wide at upper surface). A score of 1 was given for each paw which remained in position for 15 seconds, giving each rat a maximum score of 4. The duration the paw remained in position was recorded (maximum latency score 15 seconds on each trial) and total latency for paw withdrawal was determined giving each rat a maximum total latency of 60 seconds.

Statistical analysis was performed by a qualified statistician. The total catalepsy score and total latency were compared to vehicle and haloperidol by exact Wilcoxon rank sum tests. Raw means and standard errors were calculated. p<0.05 was the level used for statistical significance (n = 8/group).

**SUPPLEMENTAL FIGURES**

**Figure S1. Dopamine levels in freely moving rats**

**A.**
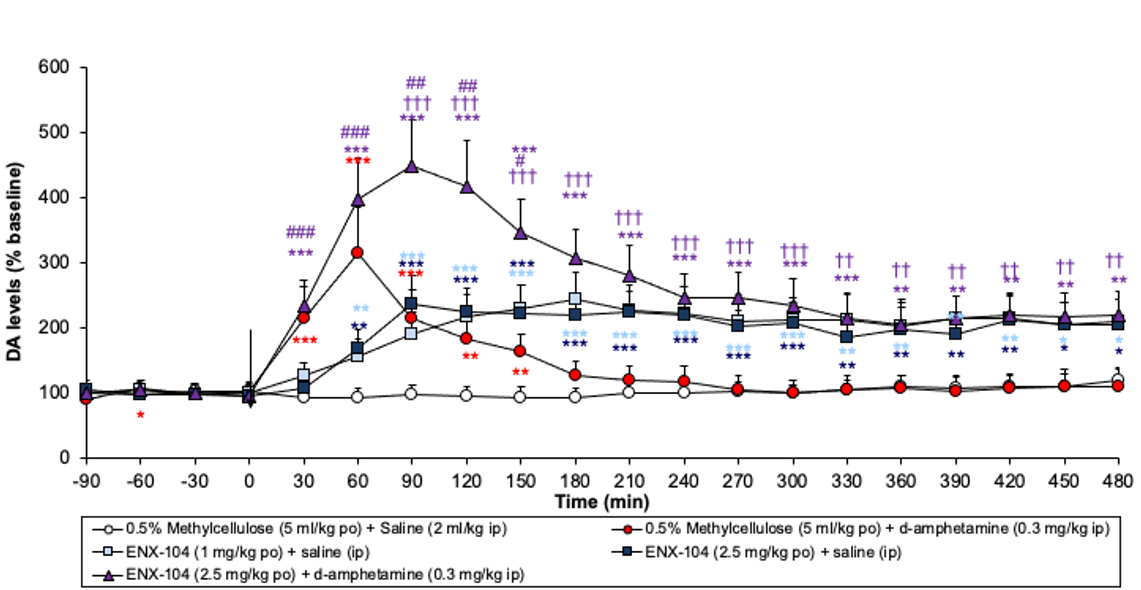
**Nucleus accumbens**

**B. Prefrontal cortex**

Timecourse of dopamine (DA) levels in the (A) nucleus accumbens and (B) prefrontal cortex of freely moving rats. ENX-104 was orally administered at 1 mg/kg (light blue squares) and 2.5 mg/kg (dark blue squares) dose levels and compared to vehicle (0.5% Methylcellulose) treated animals (white cirlces). Arrow denotes start of dose (timepoint 0). D-amphetamine (0.3 mg/kg IP, red circles) served as a positive control. Results are adjusted means (from log-transformed data) ; n=7-8/group. SEMs are calculated from the residuals of the statistical model. ENX-104 treated groups were compared to vehicle by William’s test, other comparisons to vehicle were using the multiple t test. Differences vs. vehicle (per time point) were considered statistically significant at *p < 0.05, **p < 0.01, ***p < 0.001 ; vs. ENX-104 at #p < 0.05, ##p < 0.01, ###p < 0.001 ; vs. d-amphetamine at †p < 0.05, ††p < 0.01, †††p < 0.001.

**Figure S2. Probabilistic reward task**

**
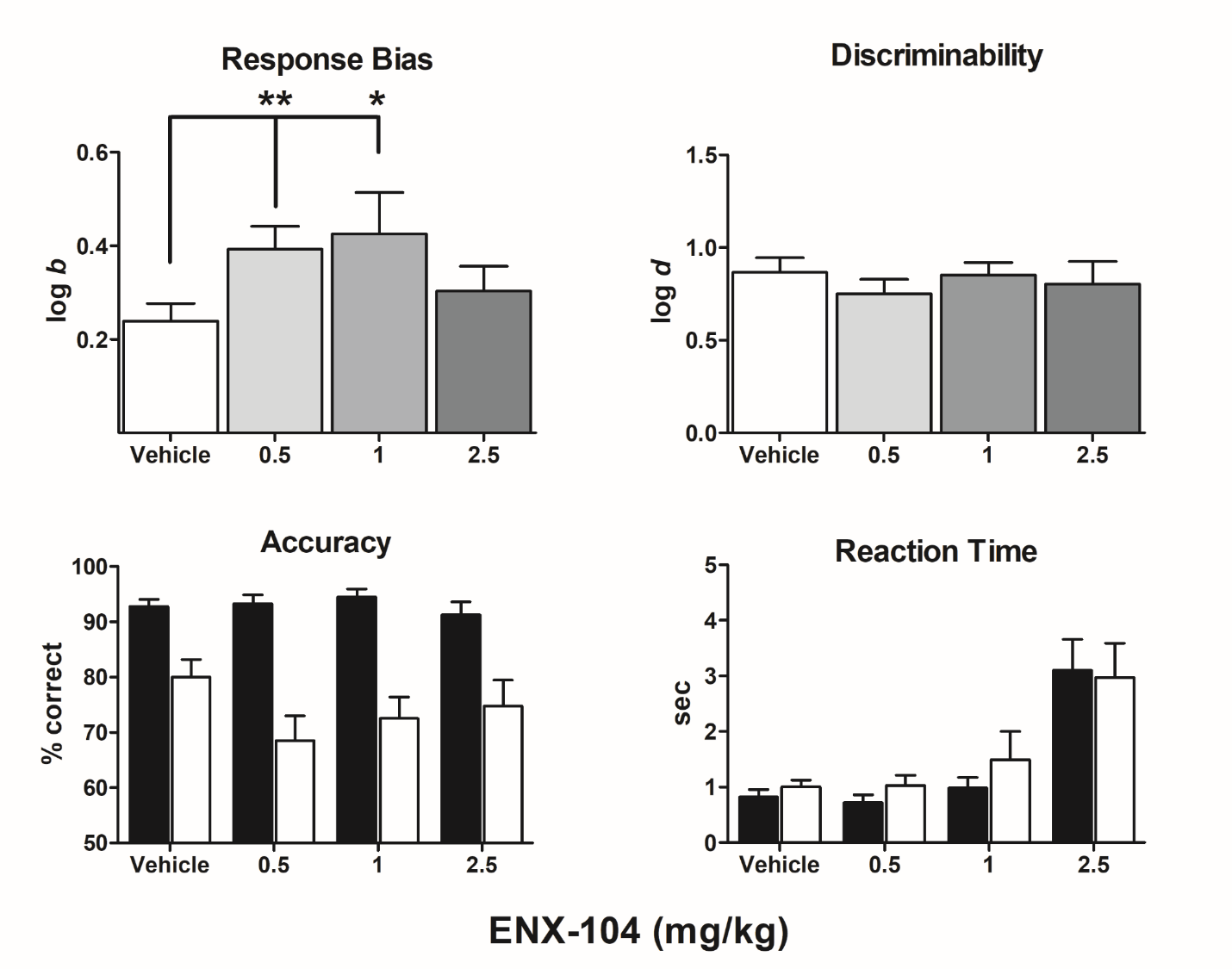
A. Discriminability (log *d*) in the PRT**

**ENX-104 (mg/kg)**

**B. Discriminability (log *d*) in the PRT**


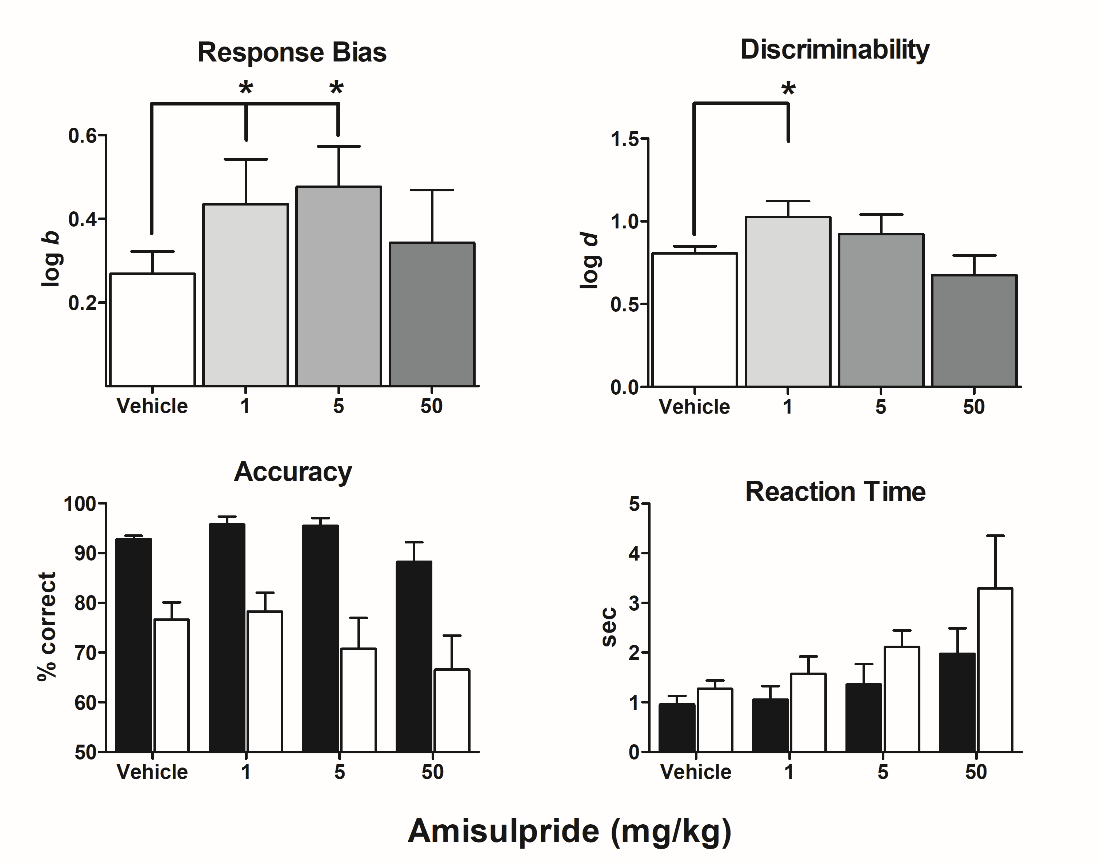


**Amisulpride (mg/kg)**

**C. Effect sizes for response bias (log *b*) in ENX-104 treated groups in the PRT**


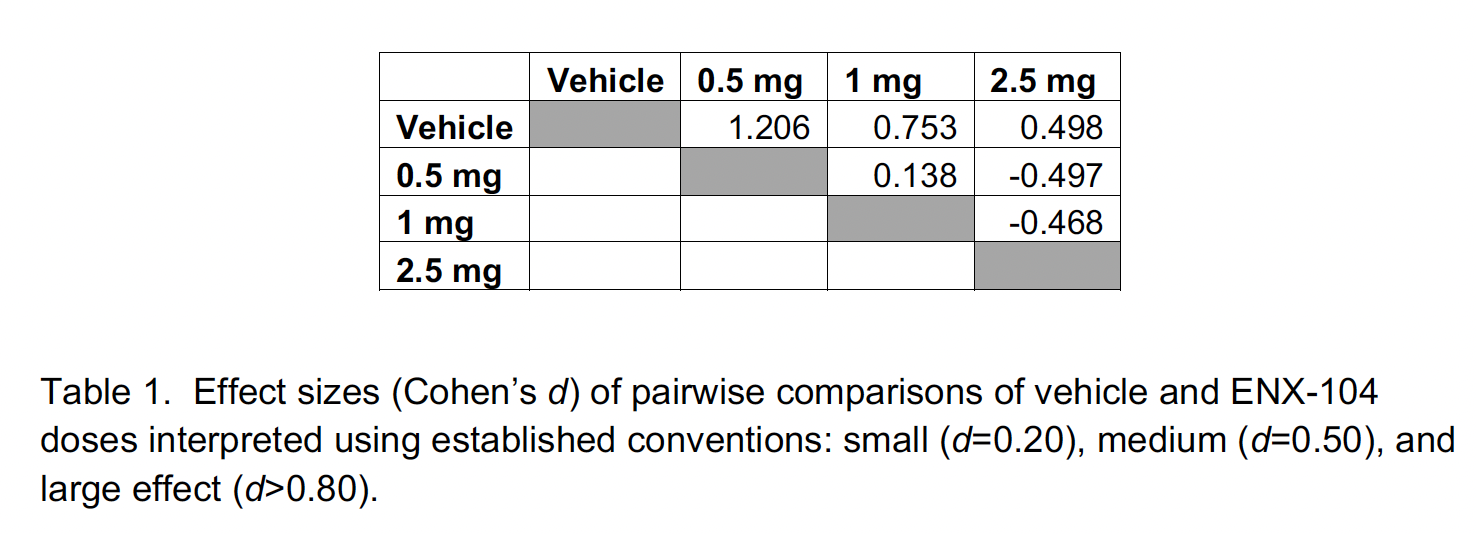


(A, B) Log d values in the PRT from ENX-104 and amisulpride treated animals. Data are presented as means + SEM. *p<0.05. (C) Log *b* effect sizes (Cohen’s *d*), pairwise comparisons of Vehicle vs. ENX-104-treated groups (at listed doses).

**Figure S3. Conditioned avoidance response assay**

1.
2.

(A) Percent avoidance response (associated with antipsychotic activity) and (B) escape failures (associated with potential motor impairment) in the CAR in orally administered ENX-104 (gray bars) (0.5 mg/kg, 2.5 mg/kg, 5 mg/kg) and control groups, administered vehicle (white bar) or risperidone (0.5 mg/kg ip, red bar). Data are presented as means + SEM. * p<0.05, ***p<0.001.

**Figure S4: Catalepsy**

1.

Catalepsy in rats was measured as (A) mean catalepsy score calculated as a sum of observations in each limb (maximum score possible is 4) and (B) latency to withdraw. Animals were orally administered ENX-104 (gray bars) at three dose levels (0.5 mg/kg, 2.5 mg/kg, 5 mg/kg, testing at 4h post dose) and control groups, administered vehicle (white bar) or haloperidol (0.5 mg/kg ip, red bar). Data are presented as means + SEM. * p<0.05.
